# A multifaceted assessment of strigolactone GR24 and its derivatives: from anticancer and antidiabetic activities to antioxidant capacity and beyond

**DOI:** 10.3389/fmolb.2023.1242935

**Published:** 2023-10-26

**Authors:** Agata Pyrzanowska-Banasiak, Tugba Boyunegmez Tumer, Bożena Bukowska, Anita Krokosz

**Affiliations:** ^1^ Department of Biophysics of Environmental Pollution, Faculty of Biology and Environmental Protection, University of Lodz, Lodz, Poland; ^2^ Department of Molecular Biology and Genetics, Faculty of Arts and Science, Canakkale Onsekiz Mart University, Canakkale, Türkiye

**Keywords:** strigolactones, GR24, mammalian cells, antioxidants, antiangiogenic activity, cancer

## Abstract

**Background:** Strigolactones are signaling molecules produced by plants, the main functions are the intracorporeal control of plant development and plant growth. GR24 strigolactone is one of the synthetic strigolactones and due to its universality and easy availability, it is a standard and model compound for research on the properties and role of strigolactones in human health.

**Purpose:** In this research work, the impact of mainly GR24 strigolactone on the human body and the role of this strigol-type lactone in many processes that take place within the human body are reviewed.

**Study design:** The article is a review of publications on the use of GR24 strigolactone in studies from 2010–2023. Publications were searched using PubMed, Elsevier, Frontiers, and Springer databases. The Google Scholar search engine was also used. For the review original research papers and reviews related to the presented topic were selected.

**Results:** The promising properties of GR24 and other strigolactone analogs in anti-cancer therapy are presented. Tumor development is associated with increased angiogenesis. Strigolactones have been shown to inhibit angiogenesis, which may enhance the anticancer effect of these γ-lactones. Furthermore, it has been shown that strigolactones have anti-inflammatory and antioxidant properties. There are also a few reports which show that the strigolactone analog may have antimicrobial and antiviral activity against human pathogens.

**Conclusion:** When all of this is considered, strigolactones are molecules whose versatile action is their undeniable advantage. The development of research on these phytohormones makes it possible to discover their new, unique properties and surprising biological activities in relation to many mammalian cells.

## 1 Introduction

Strigolactones (SLs) are signaling molecules produced by plants that have been classified as plant hormones, or phytohormones. The main function of these molecules is the intracorporeal control of plant development, they are responsible for plant growth. Strigolactones directly affect the growth of root and root hair elongation, while inhibiting the secondary development of shoots. In addition, along with auxins, they regulate the aging process of leaves, stem growth, and seed germination (Zwanenburg and Pospíšil, 2013). Strigolactones, which are components of the root exudate, act symbiotically and initiate interactions between plants and soil microorganisms. This function of strigolactones enabled their discovery in 1966. It was the first time that two strigolactones were isolated—strigol and strigyl acetate—from the exudate of cotton roots (*Gossypium hirsutum*). They stimulate the germination process of the Striga plant. Strigolactone, the commonly used name for these phytohormones, was derived from the name of the Striga plant (Xie et al., 2010).

Strigolactones are sesquiterpene lactones that belong to carotenoids. They arise through the breakdown of double bonds in carotenoid polyene, yielding structurally and functionally diverse products classified as apocarotenoids. These metabolites include plant hormones, such as abscisic acid (ABA) and strigolactones, as well as many other latterly determined substances exhibiting regulatory function.

**FIGURE 1:**
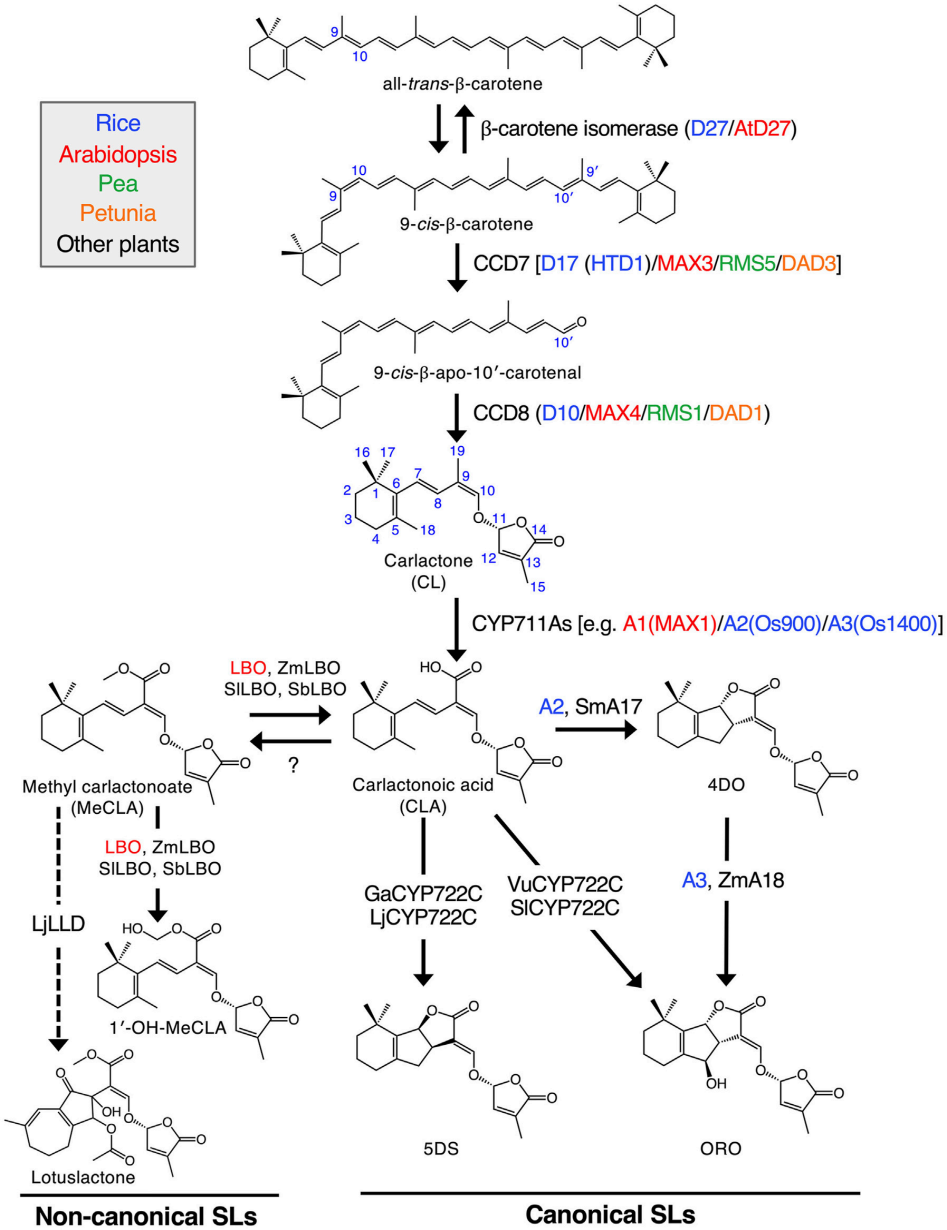
Proposed SL biosynthetic pathway. Sequential reactions catalyzed by plastid‐localized D27, CCD7 and CCD8 enzymes produce CL from all‐trans‐β‐carotene. CL is further oxidized by the CYP711A family to yield CLA. Some members of both the CYP711A and CYP722C families can produce a canonical SL, ORO, from CLA. On the other hand, GaCYP722C and LjCYP722C are involved in the production of a strigol‐type canonical SL, 5DS. For non‐canonical SLs, MeCLA was shown to be synthesized from CLA in Arabidopsis. It has recently been demonstrated that Arabidopsis, tomato, maize and sorghum LBOs convert MeCLA into 1′‐OH‐MeCLA and CLA. Also, LjLLD, encoding a novel 2OGD, was shown to be involved in the biosynthesis of a non‐canonical SL, lotuslactone. It should be noted that the substrates of LjCYP722C and LjLLD have been unknown. Enzymes of rice, Arabidopsis, pea, petunia and other plants are shown in blue, red, green, orange and black, respectively. Solid arrows indicate the confirmed pathways, and the pathways with dashed arrows have not been fully established. Zm, Zea mays (maize); Sm, Selaginella moellendorffii; Vu, Vigna unguiculata (cowpea); Sl, Solanum lycopersicum (tomato); Ga, Gossypium arboreum (cotton); Lj, Lotus japonicus; Sb, Sorghum bicolor “Reproduced with permission; from Mashiguchi et al. (2021), doi.org/10.1111/tpj.15059.”

The biosynthetic pathway for the formation of SLs in rice, Arabidopsis, pea, petunia and other plants is presented in Figure 1. SLs are mainly formed in plant roots and are transported to shoots. However, to a small extent, they can also be formed in shoots as a result of the transport of intermediates to shoots (Mashiguchi et al., 2021).

**FIGURE 2:**
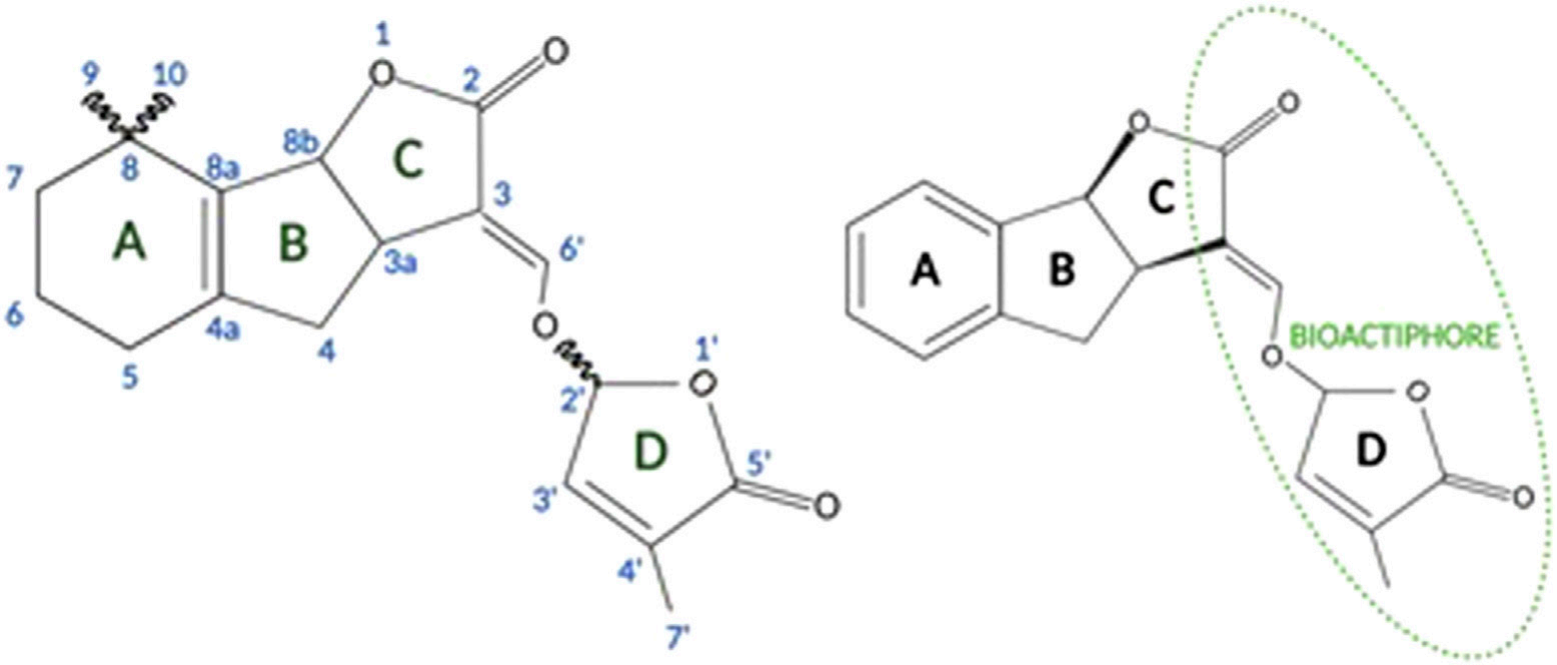
General structure of strigolactones (left panel). Synthetic GR24 strigolactone [based on Zwanenburg and Pospíšil (2013)] (right panel; created with BioRender.com).

The structure of canonical strigolactones consists of four rings, the core is ABC rings, while the D ring is connected to the core by an enol-ether bridge (Figure 2). Structurally, the ring D and an enol-ether bond constitute a conserved region that occurs naturally in all strigolactones. Because of their complex structures, the isolation of strigolactones from natural sources to a desired purity, as well as their organic synthesis on a multigram scale has been challenging. However, the identification of the D-ring as a major pharmacophore responsible for the biological activity of the molecule gave grounds for possible modifications of natural strigolactones by attaching other groups to the butanolide D-ring through the enol-ether bridge. This enabled the synthesis of analogs of natural strigolactones with simpler structures (Rasmussen et al., 2013).

The structure of the naturally occurring strigolactones and the synthesis pathways of strigolactone analogs are reviewed in the excellent papers of Prof. Prandi and her coworkers (Prandi et al., 2021; Dell’Oste et al., 2021).

The possibility of obtaining analogs of natural strigolactones allowed great progress in the identification of the activities of strigolactones in mammalian cells and their potential role in human health. The obtained analogs that have bioactivity like their natural analogs are characterized by slightly lower biological activity and higher stability (Pollock et al., 2012; Yang et al., 2022). One of the synthetic analogs of strigolactones is GR24 which is a representative analog of strigol and, due to its universality and easy availability, it is a standard and model compound for research on the properties and role of strigolactones (Halouzka et al., 2018).

The aim of this review was to discuss in detail the mechanisms of action of GR24 and its derivatives and to indicate the most interesting, in our opinion, pathways modified by SLs, i.e., angiogenesis, glucose metabolism and the antioxidant activity of SLs. Finally, the antiviral properties of SL analogs in human fibroblasts were also shown.

## 2 Review methods

The article is a review of publications on the use of GR24 strigolactone in studies from 2010–2023. The purpose of this research work is to collect knowledge on the effect of GR24 on the human body, as well as to assess the role of this strigolactone in many processes taking place within the human organism. Publications were searched using PubMed, Elsevier, Frontiers, and Springer databases. The Google Scholar search engine was also used. For the review original research papers and reviews related to the presented topic were selected. The graphical abstract summarizing the research is provided in the Supplementary Material.

## 3 Mechanisms of anticancer activity of GR24 and other SL analogs

Today, cancer is recognized as one of the major deadly diseases worldwide. It happens more and more often that traditional methods of treatment, i.e., chemotherapy, apart from several negative effects on the body, is becoming less and less effective. Hence the need to create new treatment regimens in which therapeutic drugs will be safe for normal cells and their action will be directed towards cancer cells.

**FIGURE 3:**
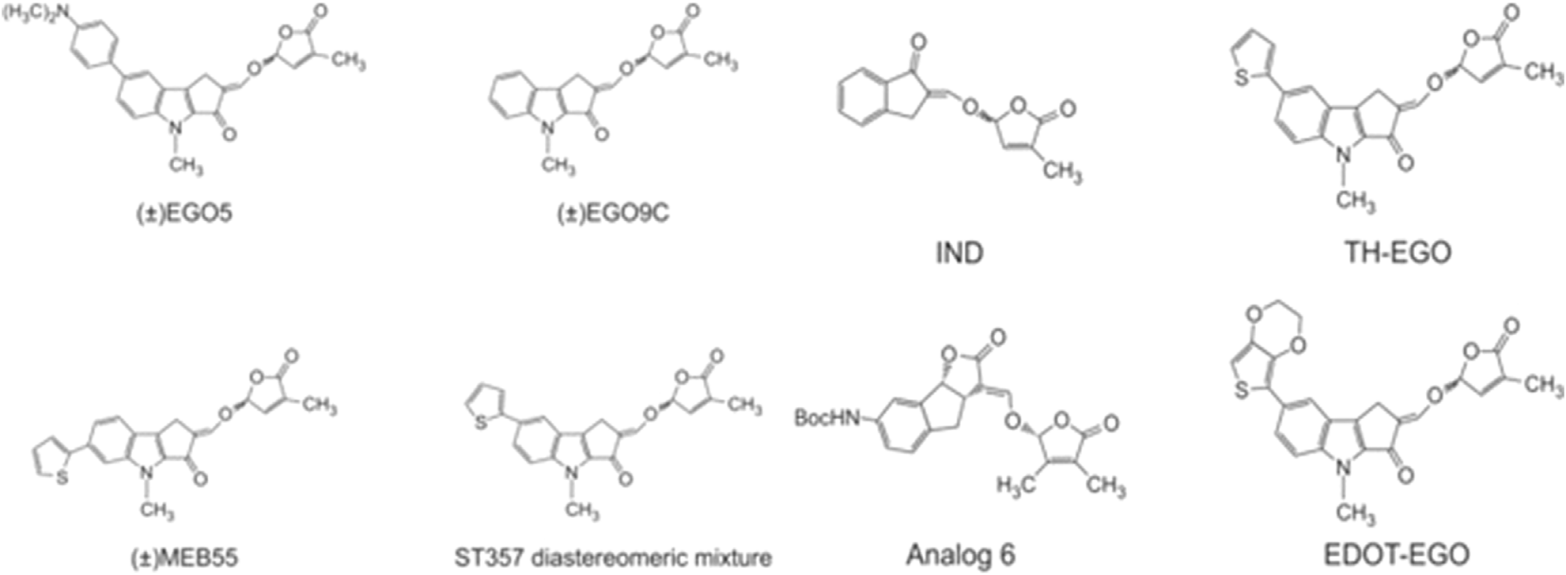
Chemical structures of strigolactones [based on Pollock et al. (2012), Biolatti et al. (2020), Antika et al. (2022), and Yang et al. (2022)].

Pollock et al. (2012) investigated the effect of synthetic analogs of strigolactones (Figure 3), including diastereomeric mixture of GR24 (Figure 2, right panel), on several tumor lines. The researchers used the following cancer cell lines: MCF-7 (hormone-dependent breast adenocarcinoma), T47D (human breast carcinoma), MDA-MB-231 (triple negative breast adenocarcinoma), MDA-436 (triple negative breast cancer), and the normal line BJ (human fibroblasts). It was shown that after 10 days of monitoring cell growth, GR24 used at a dose range 0.5–10 ppm significantly reduced cell growth in all tumor lines compared to controls. IC_50_ concentrations were—5.2 ppm (17.2 μM), for MDA-MB-436 cells and 5.7 ppm (18.8 μM) for both MDA-MB-231 and MCF-7 cells. Moreover, normal cells of the BJ line did not show a significant reduction in growth over the time tested. It was also checked how GR24 influences the cell cycle of the tested cells. Cytometric analysis of apoptosis has shown that in the case of MCF-7, MDA-MB-231 and MDA-MB-436 cells, the use of GR24 at a concentration of 0.5, 5, and 10 ppm shows a concentration-dependent increase in the percentage of cells in the G2 phase and M and a concomitant decrease in the percentage of cells in the G1 phase. In contrast, no cell cycle changes were observed in the non-cancerous MCF10A cell line. The highest concentration of GR24 caused a significant increase in the apoptotic fraction of cells (sub-G1). Researchers highlighted the ability to limit the growth of cells in the mammosphere, i.e., a culture enriched with tumor-initiating cells or cancer stem cells. Enriched cultures were shown to be more sensitive to strigolactone than cells in the monolayer. GR24 initiated the disintegration of the mammosphere and at the same time reduced its growth, which induced a reduction in the viability of the entire culture (Pollock et al., 2012).

In 2014, the same research group (Pollock et al., 2014) expanded its research towards the anti-cancer role of strigolactones. The purpose of the research was to achieve information on the mechanism responsible for the apoptosis induced by strigolactones. Thus, analogs of natural strigolactones were used such as EGO5, EG9C, ST357, ST362 (aka EDOT-EGO) and MEB55 (chemical structures in Figure 3). It has been shown that strigolactone analogs affect the arrest of the cell cycle in the G2/M phase by repressing cyclin B expression (Pollock et al., 2014). The Cyclin B1/Cyclin-Dependent Kinase 1 (Cdk1) complex is required for cell transition from the G2 phase to mitosis (Jang et al., 2016). 24 h treatment of cell lines with strigolactone, in addition to reducing the level of cyclin B, decreased the expression of Cdc25C phosphatase, which is accountable for the phosphorylation, and thus activation of the Cdk1 protein. Strigolactones also play an important role in the progression of apoptosis and cell cycle arrest due to their effect on the signaling pathway of Mitogen Activated Protein Kinase (MAPK) (Pollock et al., 2014). N-terminal C-Jun kinase (JNK) and p38 kinase activated by the stress factor promote apoptosis. Whereas Extracellular Regulated Kinase (ERK1/2) is responsible for the signaling cascade stimulated by cell growth factor (Yue and López, 2020). Pollock et al. (2014) showed that cells treated with strigolactone analogs exhibited increased expression and phosphorylation of JNK and p38 kinases with a simultaneous decrease in ERK1/2 kinase activity.

The most active analog in inhibition of cancer cell growth was EDOT-EGO for which the IC_50_ values varied from 5 to 20 μM dependent on tumor cell line. The least active one was EGO9C. The differences between these analogs are in the A-ring substituents. EGO9C has no A-ring substituents, while the most powerful analog EDOT-EGO has the bulky substituent at the position 7 of the A-ring. In contrast, GR24 has no substituents on the A-ring, and compared to its analogs, it also has an unmodified B-ring. However, its biological activity is comparable to the most active EDOT-EGO analog.

Moreover, the group showed that the blocking of the cells by strigolactone analogs activate Heat Shock Proteins (HSPs) and many cytokines. The action of strigolactones on cell lines also induces the expression of pro-apoptotic genes while reducing the expression of the ALDH1 marker, a survival factor that is crucial for the viability and self-regeneration of stem cells (Pollock et al., 2014). These studies allowed to develop knowledge of the mechanisms of interaction of strigolactones with cells and initiated the use of strigolactones in a broader spectrum of research on their anticancer activity.

The latest research by Antika et al. (2022) demonstrated the ability of SL analogs to inhibit the development of human brain tumor cell lines. Consequently, at the end of the 48-h incubation period, the IC_50_ obtained for Gr24 in A172 glioblastoma cells was 60 and 26 µM in the U87 glioblastoma cell line. However, these values decreased to 1.1 µM for the indanone-derived strigolactone (IND- Figure 3) analog which is structurally a quite different derivative of GR24 and to 14 µM for EGO10. IND is a synthetic SL analog derived from indanone with A, C, and D ring systems (Mwakaboko and Zwanenburg, 2011), and this study was the first to show its potent cytotoxic properties. In addition to inhibiting cancer cell proliferation, IND and EGO10 induced apoptosis, G1 cell cycle arrest, and expression of Bax/Caspase-3 genes, while negatively regulating Bcl-2 expression at low concentrations. The results presented these two novel pharmacophores as promising antiglioma agents that should be tested through *in vivo* studies (Antika et al., 2022).

Yang et al. (2022) decided to investigate the ability of quite a lot of strigolactone stereoisomers to inhibit the development of colorectal cancer. After screening studies, they chose a synthetic analog of GR24 obtained by chemical synthesis based on Zhang’s dynamic kinetic resolution strategy as the most potent cytotoxic agent (Zhang et al., 2014). An enantiomerically pure analog of GR24 named as Analog 6, presented in Figure 3 with IUPAC name tert-butyl-((3aS,8bR,E)-3-((((R)-3,4-dimethyl-5-oxo-2,5-dihydrofuran-2-yl)oxy)methylene)-2-oxo-3,3a,4,8b-tetrahydro-2H-indeno[1,2-b]furan-7-yl)carbamate was selected for the detailed study. The HCT116 and SW620 colon cancer cell lines and a normal human colon mucosal epithelial cell line NCM460 were used. Colony formation inhibition in colon cancer cell lines has been shown, while not having this effect in normal NCM460 cells. Analog 6 induced apoptosis in a dose-dependent manner in colon cancer cells. Moreover, Analog 6 was investigated to determine how it influences the activity of caspase-3, caspase- 8 and Poly (ADP-ribose) Polymerase 1 (PARP1), factors that are crucial in initiating the process of programmed cell death. The use of Analog 6 caused a statistically significant increase in the activity of both caspases and the PARP1 enzyme in HCT116 and SW620 cells, while this effect was not observed in the NCM460 line. These results confirmed that Analog 6 can selectively induce cytotoxicity in colorectal cancer cells by initiating apoptosis and interfere with the cell cycle by blocking cells in the S phase.

The next stage of the analysis was to investigate the phenomenon of autophagy. Autophagy is a process of self-degradation aimed at removing damaged cell organelles, abnormal or damaged proteins, and the removal of intracellular pathogens. So far, three types of autophagy have been detected: chaperone-dependent autophagy, micro-autophagy, and macro-autophagy. The forms differ in molecular mechanism, but their purpose is to deliver cytoplasmic material to the lysosome for its degradation (Glick et al., 2010). Yang et al. (2022) in their research on the phenomenon of Analog 6-induced autophagy, focused on the determination of Light Chain 3-phosphatidylethanolamine Conjugate (LC3-II) since it is a specific marker of autophagy, as it is found on both the inner and outer surfaces of the autophagosome. The use of Analog 6 at a relatively low concentration (approximately 5 µM) caused an increase in EGFP-LC3B puncta in HCT116 and SW620 cells, which was not observed in NCM460 cells. The following autophagy markers were determined through Western blot analysis: LC3B-II, p62 receptor, and Lysosomal-Associated Membrane Protein 1 (LAMP1). Analog 6 showed a significant increase in the level of these markers in HCT116 and SW620 cells, but not in NCM460 cells. The images obtained by TEM (Transmission Electron Microscopy) show an increased number of autophagic vacuoles in HCT116 cells treated with Analog 6 compared to cells treated with DMSO alone. The results obtained showed that there is colocalization of the autophagosome with mitochondria in HCT116 cells.

To check whether Analog 6 is responsible for mitophagy, the levels of PINK1 and p-Parkin were determined. PINK1 and p-Parkin are involved in a common pathway that regulates mitophagy. PINK1 detects the loss of mitochondrial membrane potential, leading to Parkin activation and its transport to damaged organelles (Chen et al., 2020). Yang and his team determined the levels of PINK1 and Parkin in Analog 6 treated cells, Western blot analysis showed that Analog 6 in HCT116 cells increased Parkin but did not affect PIKN1 levels. These results demonstrate that Analog 6 selectively inhibits mitophagy. The immunofluorescence test showed that Analog 6 blocks the autophagosome-lysosome fusion in HCT116 cells. To confirm the therapeutic potential of Analog 6, the mouse model with HCT116 xenograft was used. Analog 6 at a dose of 50 and 100 mg/kg was used in the study, immunohistochemical analysis and determination of the apoptosis marker—caspase-3 and the autophagy markers—p62 and LC3B-II showed that the use of this compound significantly leads to necrotic changes in tumor cells and leads to an increase in the level of markers. At both doses of Analog 6, attenuation of tumor growth was observed with low toxicity in mice. The results obtained confirm the therapeutic potential of the strigolactone analog and emphasize its significant anticancer properties (Yang et al., 2022).

Taking together, as we can see in Table 1 screening studies of the cytotoxicity of SLs have shown that the cytotoxic activity of SLs is associated with modifications in the A and D rings, while the modification of the B ring is of negligible importance for the cytotoxicity of SLs. Moreover, toxic effect of SLs is strictly dependent on cancer cell line and incubation time.

**TABLE 1:** IC_50_ values of strigolactone analogs in mammalian cell lines.

Strigolactone analogs	Cell line	Incubation time	IC_50_	References
GR24	BJ fibroblasts	7 days	>>10 ppm	Pollock et al. (2012)
	MCF7		5.7 ppm (18.8 μM)	
	MDA-MB-231		5.7 ppm (18.8 μM)	
	MDA-MB-436		5.2 ppm (17.2 μM)	
EGO5	MCF10A	72 h	>15 ppm	
	MCF-7		17.5 ppm	
	T47D		8.8 ppm	
	MDA-MB-231		7.5 ppm	
EGO9C	MCF10A		>15 ppm	
	MCF-7		17.3 ppm	
	T47D		>10 ppm	
	MDA-MB-231		>10 ppm	
	MDA-MB-436		>10 ppm	
ST357	MCF10A		>15 ppm	
	MCF-7		>20 ppm	
	T47D		>10 ppm	
	MDA-MB-231		5.0 ppm	
ST362	MCF10A		>15 ppm	
	MCF-7		8.1 ppm	
	T47D		8.6 ppm	
	MDA-MB-231		2.9 ppm	
	MDA-MB-436		5.9 ppm	
MEB55	MCF10A		>15 ppm	
	MCF-7		>12.8 ppm	
	T47D		5.0 ppm	
	MDA-MB-231		3.9 ppm	
	MDA-MB-436		8.3 ppm	
EG5	PC3	72 h	>15 ppm	Pollock et al. (2014)
	DU145		13 ppm	
	LNCaP		>15 ppm	
	HT-29		>15 ppm	
	HCT116		>15 ppm	
	SW480		>15 ppm	
	A549		4.7 ppm	
	U20S		3.9 ppm	
EG9C	PC3		>15 ppm	
	DU145		>15 ppm	
	LNCaP		11.5 ppm	
	HT-29		>15 ppm	
	HCT116		>15 ppm	
	SW480		>15 ppm	
	A549		15.5 ppm	
	U20S		4.5 ppm	
	BJ Fibroblasts		>20 ppm	
ST357	PC3		5.0 ppm	
	DU145		8.5 ppm	
	LNCaP		11.4 ppm	
	HT-29		>15 ppm	
	HCT116		>15 ppm	
	SW480		>15 ppm	
	A549		4.9 ppm	
	U20S		4.5 ppm	
	BJ Fibroblasts		>20 ppm	
ST362	PC3		10.3 ppm	
	DU145		7.5 ppm	
	LNCaP		2.5 ppm	
	HT-29		7.3 ppm	
	HCT116		5.2 ppm	
	SW480		2.9 ppm	
	A549		5.2 ppm	
	U20S		2.8 ppm	
MEB55	PC3		8.8 ppm	
	DU145		10.8 ppm	
	LNCaP		2.99 ppm	
	HT-29		8.2 ppm	
	HCT116		10.4 ppm	
	SW480		9.7 ppm	
	A549		5.1 ppm	
	U20S		2.7 ppm	
	BJ Fibroblasts		>20 ppm	
GR24	A172	24 h	96.9 ± 6.8 µM	Antika et al. (2022)
		48 h	59.9 ± 3.4 µM	
		72 h	38.0 ± 9.0 µM	
	U87	24 h	69.5 ± 11.3 µM	
		48 h	26.2 ± 2.3 µM	
		72 h	25.1 ± 2.9 µM	
	HUVEC	24 h	62.7 ± 1.1 µM	
		48 h	40.6 ± 5.0 µM	
		72 h	31.1 ± 1.1 µM	
EGO10	A172	24 h	28.5 ± 4.3 µM	
		48 h	15.3 ± 1.8 µM	
		72 h	17.1 ± 0.2 µM	
	U87	24 h	64.8 ± 17.0 µM	
		48 h	14.0 ± 3.5 µM	
		72 h	17.5 ± 3.2 µM	
	HUVEC	24 h	37.7 ± 1.2 µM	
		48 h	20.1 ± 1.2 µM	
		72 h	21.6 ± 1.1 µM	
IND	A172	24 h	2.5 ± 1.0 µM	
		48 h	2.8 ± 0.2 µM	
		72 h	0.8 ± 0.1 µM	
	U87	24 h	17.0 ± 0.9 µM	
		48 h	1.1 ± 0.1 µM	
		72 h	1.2 ± 0.2 µM	
	HUVEC	24 h	4.4 ± 1.3 µM	
		48 h	2.1 ± 1.3 µM	
		72 h	2.9 ± 1.5 µM	
GR24	BAEC	3 days	71.99 ± 6.20 µM	Carrillo et al. (2019)
	HeLa		49.44 ± 0.51 µM	
	HL-60		42.71 ± 6.12 µM	
	HT-29		93.17 ± 6.35 µM	
	HT-1080		78.98 ± 18.4 µM	
	MDA-MB-231		86.47 ± 6.87 µM	
	U2-OS		64.8 ± 13.2 µM	
	U-87MG		72.45 ± 6.39 µM	

## 4 GR-24 in angiogenesis

**FIGURE 4:**
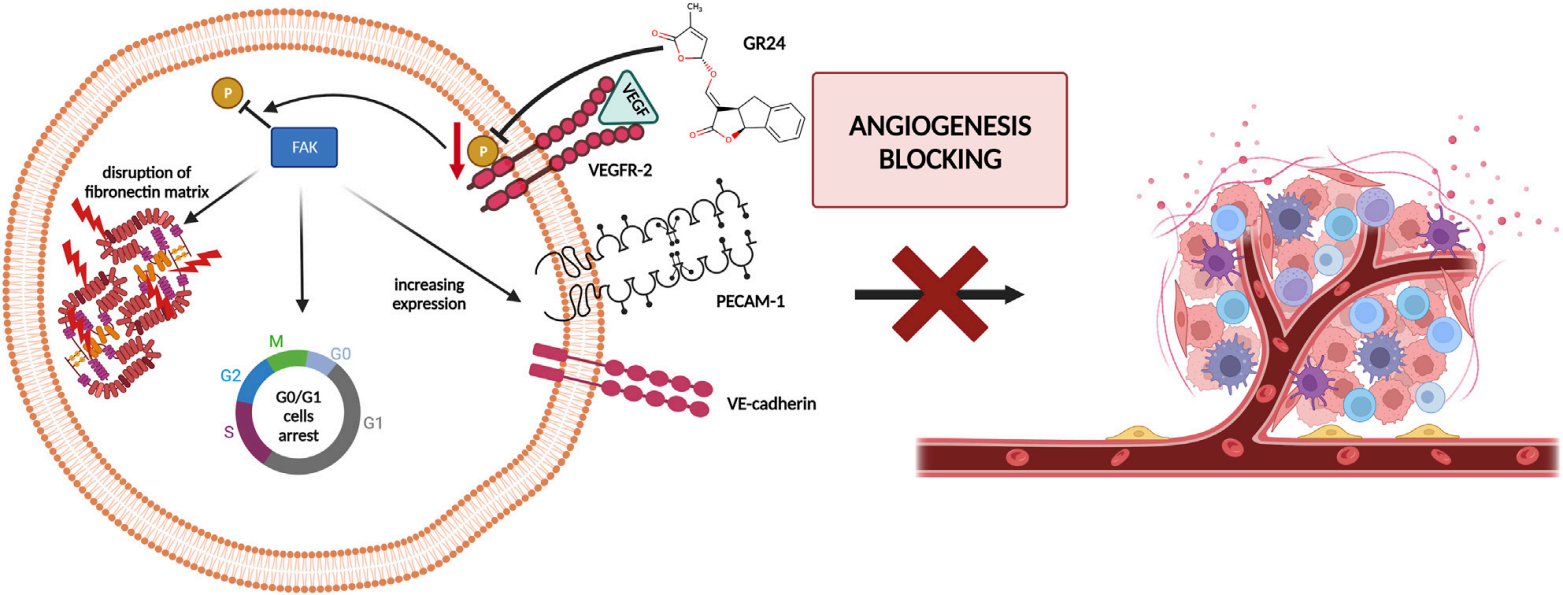
The anti-angiogenic effect of GR24 by blocking Vascular Endothelial Growth Factor (VEGF) signaling and in the end reduction of Focal Adhesion Kinase (FAK) activity [based on Carrillo et al. (2019)]; created with BioRender.com.

Research on the anticancer properties of GR24 was also conducted by Carrillo et al. (2019). The research team studied the effects of GR24 on the angiogenesis process *in vitro* and *in vivo*, GR24 disrupts the development of the chorioallantoic membrane (CAM) during chick embryo formation and inhibits the formation of distal intersegmental vessels in transgenic zebrafish. In the case of the CAM analysis, it was shown that at 25 nmol/CAM, inhibition of angiogenesis of over 90% was achieved. However, in the case of the zebrafish assay, it was shown that antiangiogenic activity is dose-dependent and at a concentration of 10 µM GR24 it is 100%. The results of *in vivo* studies led the researchers to analyze the antiangiogenic potential of GR24 against normal and neoplastic cells. For this purpose, the following cell lines were used: pooled human umbilical vein endothelial cells (HUVEC), bovine aortic endothelial cells (BAEC), glioblastoma U87MG, promyelocytic leukemia HL60, fibrosarcoma HT-1080, cervix adenocarcinoma HeLa, hepatoma HepG2, osteosarcoma U2OS, breast carcinoma MDA-MB-231, neuroblastoma SK-N-SH and adenocarcinoma HT-29. The half-maximal inhibitory concentration (IC_50_) value was determined based on the survival curves. It was observed GR24 inhibited endothelial cell proliferation with an IC_50_ of approximately 72 µM in BAEC. However, the specificity of the action of GR24 in relation to endothelial cells was not demonstrated, because the IC_50_ for various tumor cell lines fluctuated in the range of 40–90 µM GR24. Carrillo et al. decided to refer to the results presented in 2012 by Pollock et al. (2012). The influence of GR24 on the course of the BAEC endothelial cell cycle and the breast cancer cell line MDA-MB-231 was investigated. In BAEC, GR24 was observed to cause cell accumulation of cells in the G0/G1 phase without increasing the population of apoptotic cells (sub-G1 phase). In contrast, in the case of MDA-MB-231 cells, cell cycle analysis showed cell accumulation in the G2/M phase, which is in line with previous results obtained by Pollock et al. The anti-angiogenic effect of GR24 is based on the reduction of focal adhesion kinase (FAK) activity by disrupting vascular endothelial growth factor (VEGF) signaling. GR24 inhibits VEGF phosphorylation, which in turn disrupts the signaling pathway and contributes to the reduction of further activation of FAK, which is an essential factor in angiogenesis. Additionally, GR24 directly increases the resting phenotype in the cellular endothelium and prevents angiogenesis by affecting the expression of endothelial adhesion molecules, Vascular Endothelial Cell Cadherin (VE-cadherin) and Platelet Endothelial Cell Adhesion Molecule (PECAM-1) (Figure 4) (Carrillo et al., 2019). These results suggest that the strigolactone analog may have specific properties against tumor cells, making it a promising candidate for antiangiogenic tumor therapy.

## 5 The direct and indirect antioxidant capacity of GR24

The ability of plant strigolactones to influence the development of both the under and above-ground parts of plants prompted researchers to evaluate the function of these phytohormones in relation to various environmental factors, such as oxidative stress.

Direct antioxidants are short-lived small molecules with redox potential that directly capture reactive oxygen and/or nitrogen species that need to be replenished continuously. On the other hand, indirectly acting antioxidants can or cannot be redox active. They generally induce a battery of antioxidant enzyme system. A review of the up-to-date investigations presented in scientific literature indicates that SLs have an antioxidant effect through indirect action.

Phase II Xenobiotic Metabolizing Enzymes (XMEs) ability a shared Kelch Like ECH Associated Protein 1/NF-E2-related Factor 2/Antioxidant Response Element (Keap1/Nrf2/ARE) pathway are activated. These pathways lead to raised antioxidant ability and long-lived preventive effect compared to directly acting antioxidants. Numerous studies conducted in plants and mammalian cells indicated the direct and indirect antioxidant potential of GR24 (Tumer et al., 2015).

The numerous studies carried out in plants and the achievement of convergent results indicate the high antioxidant potential of GR24 strigolactone (Zhang et al., 2020; Mostofa et al., 2021; Ma et al., 2022; Zhou et al., 2022).

**FIGURE 5:**
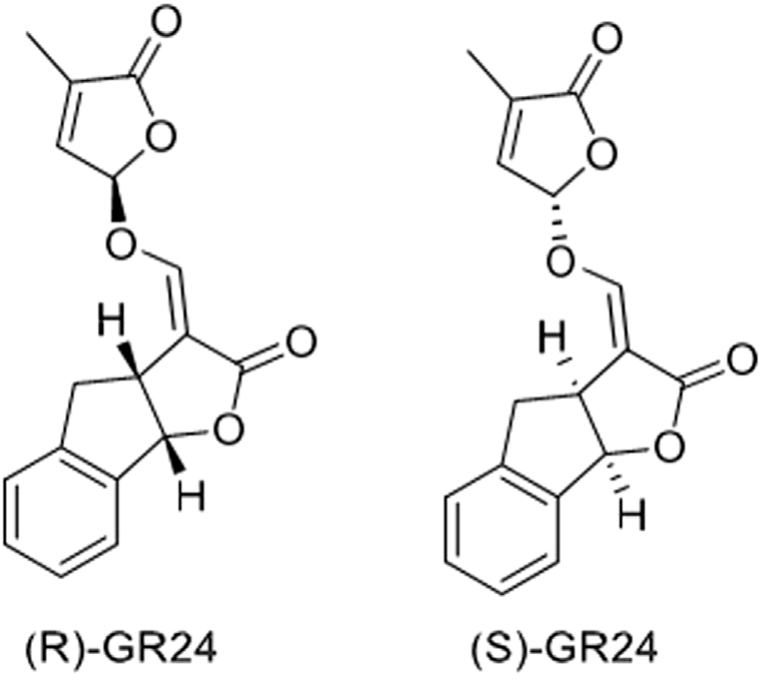
Structures of two enantiomers, (R)-GR24, IUPAC name: (3aR*,8bS*,E)-3-((((R*)-4-methyl-5-oxo-2,5-dihydrofuran-2-yl)oxy)methylene)-3,3a,4,8b-tetrahydro-2H-indeno[1,2-b]furan-2-one and (S)-GR24, IUPAC name: (3aS*,8bR*,E)-3-((((S*)-4-methyl-5-oxo-2,5-dihydrofuran-2-yl)oxy)methylene)-3,3a,4,8b-tetrahydro-2H-indeno[1,2-b]furan-2-one (Tumer et al., 2018).

**FIGURE 6:**
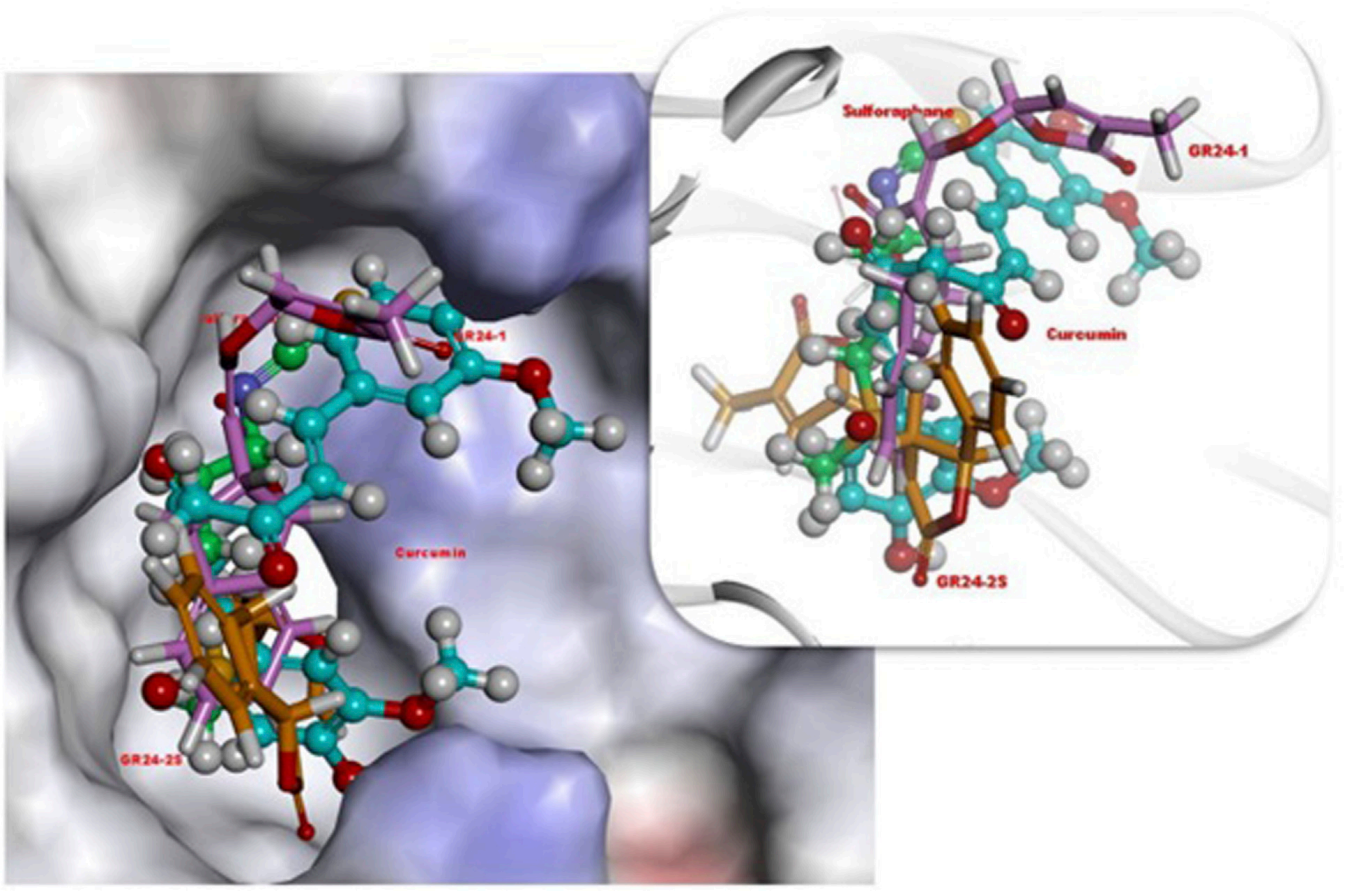
3D orientations of (R)-GR24 (light pink, stick), (S)-GR24 (orange, stick), sulforaphane (green color, ball and stick) and curcumin (light blue, ball and stick) in active site of Keap1. Reproduced with permission; from Tumer et al. (2018), doi.org/10.1016/j.compbiolchem.2018.07.014.

Tumer et al. (2018) investigated the effect of GR24 on the Nrf2 signaling pathway and the downstream cytoprotective/antioxidant enzyme system in murine Hepa1c1c7 cells, a well-established model for the identification of Nrf-2 inducers and murine Raw264.7 macrophage cells. Nrf2 is a transcription factor that regulates a wide range of catalytically active antioxidant and detoxifying response mechanisms. In addition to its role in cytoprotection, studies confirmed that induction of Nrf2 also stimulates NADPH synthesis, toxin export, and nucleotide excision repair while inhibiting cytokine-mediated inflammation (Tumer et al., 2018). The Nrf2 activity-dependent action of antioxidant target genes leads to the blockade of the NF-KB pro-inflammatory transcription factor NF-KB, which contributes to the blocking of the transcription of inflammation-promoting mediators. Therefore, Nrf2 activation weakens many pathogenic processes, which are associated with inflammation and oxidative stress, including cancer, obesity, diabetes, or neurodegenerative diseases (Vomund et al., 2017). Tumer et al. (2018) observed that GR24 caused the activation of Nrf2 signaling and increased the expression of downstream ARE-responsive genes in macrophage and hepatic cell lines. Cell exposure for 24 h to GR24 at very low doses (10–50 μM) resulted in significant induction in the mRNA expression of Heme Oxygenase-1 (HO-1) and NADPH-quinone Oxidoreductase-1 (NQO1) enzymes (at max. 64 and 58 times, respectively). The intracellular regulator of Nrf2 activity is the protein KEAP1. Nrf2 is found mainly in the cytosol, where it binds to KEAP1. KEAP1 leads to Nrf2 ubiquitination leading to degradation by the proteasomes and therefore strictly ensures a low level of Nrf2 expression (Song et al., 2021). Tumer et al. (2018) used *in silico* molecular dynamic simulations and docking procedures to determine how GR24 enantiomers, presented in Figure 5 affect the KEAP1 and Nrf2 complex. By disrupting the junction between these molecules allow Nrf2 to translocate to the nucleus and induce expression of phase II detoxification enzymes phase II. Molecular docking has shown that GR24 could interact with the 16-mer site of the KEAP1 peptide, that is, the Neh2 domain to which Nrf2 binds (Figure 6). The biological activity of GR24 is manifested by the disruption of the connection between KEAP1 and Nrf2, which allows the activity of the Nrf2 factor to increase and the activation of its antioxidant mechanism, including the activation of phase II detoxifying enzymes. Moreover, molecular docking showed that the (R)-GR24 enantiomer binds more strongly than (S)-GR24 to KEAP1. This is due to the orientation of the R enantiomer and its better fit to the active pocket of KAEP1, which is confirmed by the calculated binding energy of this enantiomer. The binding energy of (R)-GR24 is even greater than that of powerful antioxidants such as sulforaphane and curcumin. It is worth noting that natural strigolactones have the R conformation in the 2' position (Mashiguchi et al., 2021). The molecular docking results indicate that such a conformation in the 2' position determines greater biological activity.

**FIGURE 7:**
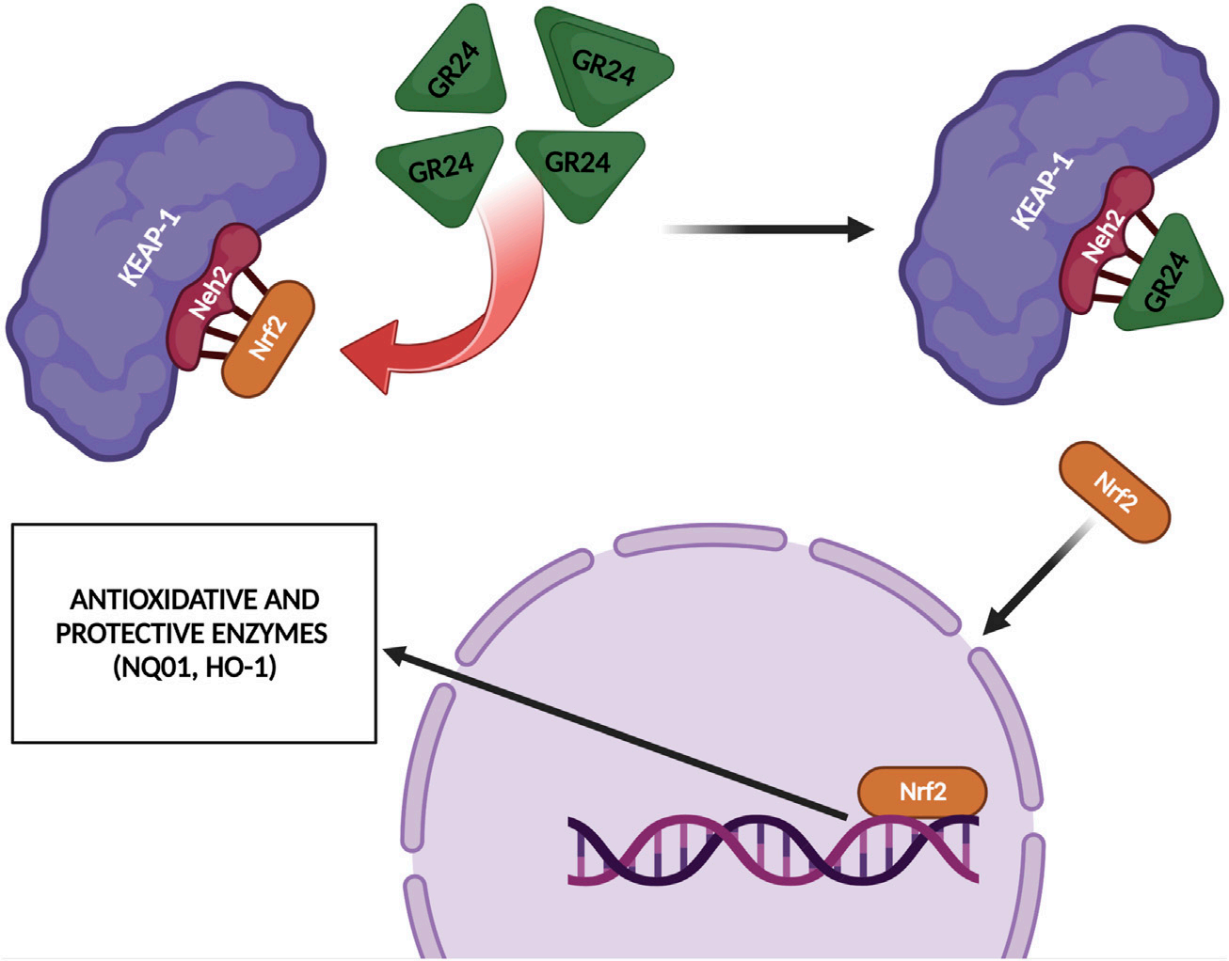
GR24 upregulated some Nrf2-related antioxidant enzymes such as HO-1 and NQO1 [based on Modi and Kokkola (2018) and Tumer et al. (2018)]; created with BioRender.com.

Modi and Kokkola (2018) also investigated the effect of GR24 strigolactone on the modulation of gene expression of Nrf2. In their research model, Modi and Kokkola (2018) used rat L6 myoblasts, which were differentiated into myotubes. The cells were then treated with 60 µM GR24 for 24 h. The analysis was based on microarrays. The results obtained allowed to observe that the stimulation of GR24 cells leads to enhanced expression of Nrf2-dependent genes, i.e., antioxidant genes Nqo1 and heme oxygenase 1, which are responsible for blocking the signaling pathway leading to inflammation formation. Moreover, GR24, by promoting the activation of the Nrf2 factor and its target genes, contributed to the enhanced activation of antioxidant defense in rat myotubes (Figure 7). These results also demonstrated the potential of GR24 to alleviate oxidative stress and the therapeutic potential of this strigolactone analog in stress-related health conditions. As mentioned above, Nrf2 is a factor that plays a key role in the regulation of the cellular response to oxidative stress (Tumer et al., 2018).

## 6 The role of GR24 in modulating inflammation

The inflammatory reaction is the response of the body’s immune system to harmful factors. These factors include pathogenic microorganisms and viruses, and inflammation can also be caused by numerous cell damage, toxic compounds, or physical factors, such as radiation, injuries, or burns. The inflammatory response is a complex process of cellular interaction at the molecular level to restore the homeostasis of the body. The effect of damage is to trigger a signaling cascade that induces reactions aimed at restoring proper functioning and healing the damage (Chen et al., 2018).

Many researchers are looking for compounds that could mitigate or completely prevent the harmful effects of inflammation on the body in various states of disease. Also, in this aspect, attempts have been made to evaluate the role and effect of the GR24 strigolactone analog on inflammation.

Modi et al. (2017) elucidated how GR24 may affect glucose metabolism and SIRT1 (Silent Information Regulator Type 1) regulation. SIRT1 is a NAD + -dependent deacetylase that could bind and deacetylate many transcription factors that promote inflammation, i.e., Nef2 (Nuclear Factor E2-related Factor 2), NF-kB (Nuclear Factor kB), PDX-1 (Pancreatic and Duodenal Homeobox Factor 1), and Forkhead Box class O (FOXO). As a result, SIRT1 can regulate oxidative stress and inflammation (Singh and Ubaid, 2020).

The use of GR24 has been shown to enhance the expression and activate SIRT1 in rat skeletal muscle cells. GR24 increased basal as well as insulin-stimulated glucose uptake. Moreover, GR24 increased the level of GLUT4 (Glucose Transporter Type 4) protein and stimulated its translocation. The analysis of the microarrays and the appropriate sets of genes showed that GR24 stimulates the biogenesis of mitochondria. Figure 9 summarizes investigations proving that GR24 may regulate glucose metabolism by activating SIRT1. Therefore, the phytohormone analog GR24 may be a hope for people with type 2 diabetes and insulin resistance (Modi et al., 2017).

Further studies by Modi and Kokkola (2018) used knowledge about the ability of GR24 strigolactone to activate SIRT1 and decided to investigate its effect in the context of adipogenesis and inflammation in adipocytes. Adipocytes are the main cells of adipose tissue; their primary task is to synthesize energy storage in the form of triglycerides—simple fats (Rutkowski et al., 2015). Obesity is a condition in which adipose tissue is rearranged, significantly changes its structure and cellular composition. This is because adipose tissue must absorb excess caloric intake. Remodeling of adipose tissue in obesity initiates chronic inflammation associated with a disturbance in the production of cytokines (Fuster et al., 2016). Studies indicate that SIRT1 can inhibit adipogenesis by reducing the expression of PPARγ (Peroxisome Proliferator-activated Receptor Gamma) and C/EBPα (CCAAT-enhancer-binding Protein Alpha), which are the main regulators of adipogenesis transcription (Majeed et al., 2021). Modi et al. (2018) observed that the use of GR24 at a concentration of 60 µM against mouse 3T3-L1 preadipocyte fibroblasts inhibited the proliferation of these cells with the simultaneous inhibition of fat accumulation during adipocyte differentiation. GR24 was also shown to activate SIRT1 and increased the NAD + level of the basic SIRT1 co-substrate. Increased SIRT1 activity led to inhibition of adipogenesis in 3T3-L1 through suppression of PPARγ and C/EBPα. TNF-α (Tumor Necrosis Factor α) -induced inflammation in adipocytes was significantly reduced by GR24 due to the ability of the phytohormone analog to reduce the activity of the pro-inflammatory factors NF-KB and interleukin 6. These results demonstrated the beneficial effect of GR24 on the reduction of inflammation in adipocytes.

**FIGURE 8:**
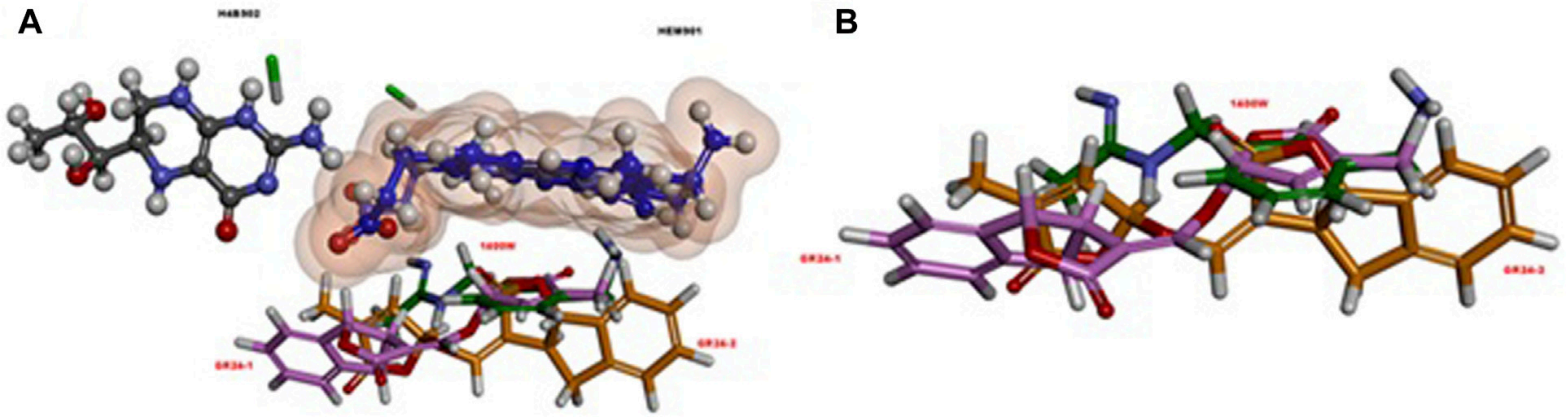
**(A)** The relative orientation of ligands, rac-GR24 (GR24-1: a light pink ball and stick; GR24-2: orange ball and stick) and 1,400 W (dark green, stick), in relation to the HEM901 (dark blue, ball and stick) and H4B902 (grey, ball and stick) groups in iNOS. **(B)** A different view, without the HEM901 and H4B902 groups in iNOS. Reproduced with permission; from Tumer et al. (2018), doi.org/10.1016/j.compbiolchem.2018.07.014.

Tumer et al. (2018) investigated the anti-inflammatory potential of GR24 in murine RAW macrophages (RAW264.7) and its effects on glucose metabolism in C2C12 myoblasts, as well as in H4IIE hepatocytes RAW264.7 macrophages were treated with bacterial lipopolysaccharide (LPS) and then assessed how treatment of GR24 cells would influence NO (nitric oxide) (Tumer et al., 2018). NO is synthesized with the participation of enzymes belonging to the family of nitric oxide synthases (NOS). NOS occurs in the body in the form of 3 isoforms. Two of them nNOS—neuronal and eNOS—endothelial are the base forms, while the expression of the third is induced, hence its name iNOS. The iNOS isoform is not permanently present in the body. It is expressed by stimulation by pro-inflammatory cytokines or LPS. After iNOS activation, NO is produced, which is a crucial signaling molecule and is involved in the fight against pathogens, which underlines its importance in the inflammatory response and activation of the response of the immune system (Cinelli et al., 2020). The use of GR24 significantly influenced the inhibition of NO formation in RAW264.7 macrophages in a dose-dependent manner. The IC_50_ of GR24 (approximately 6 µM) was determined to be comparable to the IC_50_ of a selective and irreversible iNOS inhibitor N-(3-(aminomethyl)benzyl)acetamidine (1,400 W). Furthermore, the researchers investigated how GR24 can affect the expression of the iNOS enzyme, the results showed that iNOS mRNA expression was suppressed in a dose-dependent manner with GR24, which confirmed previous observations. Tumer et al. (2018) also performed molecular dynamic simulations and docking studies against iNOS. *In silico* results confirmed the research, because more favorable values were obtained for GR24 than for the standard inhibitor iNOS 1,400 W. Figure 8 shows visualization of molecular docking of GR24 enantiomers on iNOS enzyme. As for KAEP1, the binding energy indicates a stronger interaction of (R)-GR24 with iNOS compared to (S)-GR24 or even to 1,400 W. The orientation of (R)-GR24 is better suited to enzyme ligands HEM901 and H4B902 than (S)-GR24 or 1,400 W. (R)-GR24 binds to iNOS through four hydrogen bonds while (S)-GR24 creates only three hydrogen bonds (Tumer et al., 2018).

The studies also considered the influence of GR24 on other factors related to inflammation, such as: cyclooxygenase 2, NF-kB or IL-1β. Cyclooxygenase 2 (COX-2) is responsible for the formation of prostaglandins, which are pro-inflammatory (Verburg et al., 2001).

**FIGURE 9:**
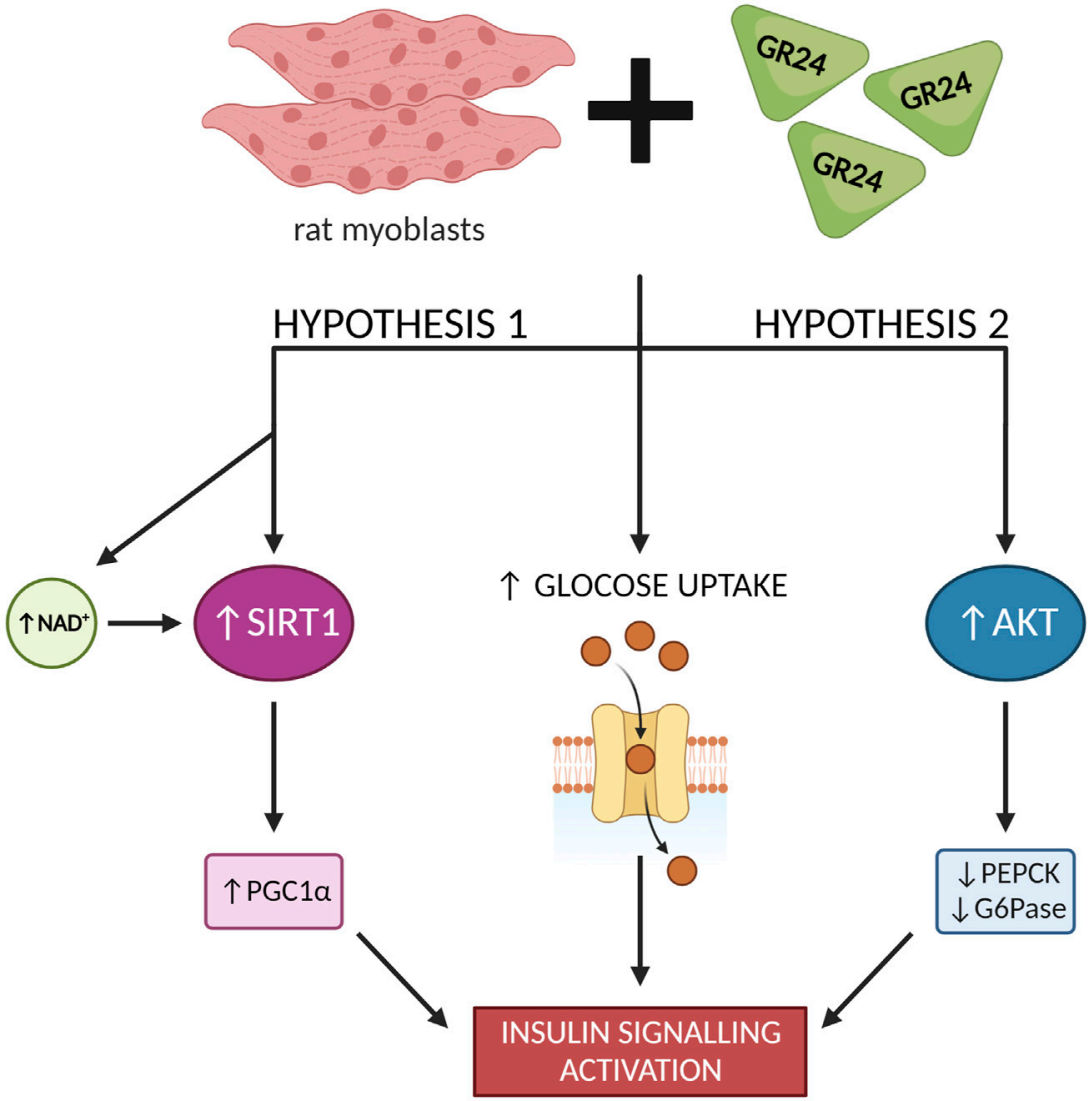
GR24 exerts a variety of effects on glucose metabolism in skeletal muscle cells [based on Modi et al. (2017) and Tumer et al. (2018)]; created with BioRender.com.

NF-kB is a transcription factor that turns on the genes necessary to activate the immune system in response to inflammation. NF-kB belongs to a family of five structurally similar factors, which include NF-kB1 (p50), NF-kB2 (p52), RelA (p65), RelB and c-Rel. Activation of NF-kB is related to the degradation of IκBα through its phosphorylation, which is caused by the multisubunit IκB Kinase Complex (IKK). The action of IKK is induced in response to various stimuli, e.g., cytokines, growth factors, mitogens, pathogen components, and stress factors. Activation of IKK results in phosphorylation of IκBα, contributing to ubiquitin-dependent destruction of IκBα in the proteasome. This results in nuclear translocation of NF-kB factor (Liu et al., 2017). However, IL-1β is a potent pro-inflammatory cytokine that is produced mainly by macrophages and monocytes in response to signaling molecules called Pathogen Associated Molecular Patterns (PAMPs) (Lopez-Castejon and Brough, 2011). Researchers showed that GR24 also inhibited COX-2 expression in a dose-dependent manner. In the case of IL1β secretion by cells treated with LPS, the addition of GR24 significantly inhibited the release of this cytokine of the order of micromolar concentrations. Instead, it significantly inhibited the expression of IL1β in doses of 10, 20, and 50 μM by 28, 30%, and 70%, respectively. Western blot analysis showed that treatment of RAW264.7 macrophages simultaneously with LPS (1 μg/ml) and GR24 (20 μM) significantly reduced the accumulation of the RelA unit (p65) in the cell nucleus, which confirms the anti-inflammatory properties of this strigolactone analog in addition to the anti-inflammatory effects of GR24 in murine macrophages. Tumer et al. (2018) also showed that GR24 inducted AKT activity in insulin-resistant skeletal muscle cells and downregulated PEPCK (Phosphatoenolpyruvate Carboxykinase) and G6Pase, which are rate-controlling enzymes of gluconeogenesis in liver cells. They proposed that AKT activation through Ser473 phosphorylation can also be one of the mechanisms for improved insulin signaling (Figure 9) in addition to SIRT1 activation, which was previously suggested as the underlying mechanism by Modi et al. (2017).

The possible interactions and therapeutic potential of GR24 have also been explored in the brain microenvironment considering inflammation-related signaling pathways and cytoprotective mechanisms. Research presenting the relevance of plant hormones to the mammalian brain is quite interesting. Many phytohormones, such as cytokines, gibberellic acids, and acetic acid were detected in the brain of laboratory rodents after dietary treatments. Therefore, they can penetrate the blood-brain barrier and tend to accumulate in the brain (Garabrant and Philbert, 2002). In addition, the mammalian brain can produce and perceive some of the plant hormones such as abscisic acid, structurally close to retinoic acid, under stress conditions (Qi et al., 2015). Furthermore, while some phytohormones have protective effects on mammalian brain cells, others may have toxic effects. For example, there were studies showing chemo-preventive effects Zeatin, a kind of cytokines, in Alzheimer’s disease (Kim et al., 2008; Choi et al., 2009). On the other hand, indole acetic acid can stimulate apoptosis in the neuroepithelium and may cause low neuronal counts and microencephaly in the fetus (Furukawa et al., 2005). A recent study showed that ABA ameliorated learning and memory problems in rats with Alzheimer’s disease by modulating PPAR receptors and the 44 PKA signaling pathway (Khorasani et al., 2019).

Very recently, the study by Kurt et al. (2020) showed the effect of strigolactones on the resident macrophages of the brain and blood brain barrier endothelium cells. Researchers used the SIM-A9 microglia cell line treated with the racemic mixture GR24 (Kurt et al., 2020). Inflammation in the nervous system is one of the body’s defense mechanisms to protect and restore the brain to normal function after an infection or injury. However, chronic inflammation can induce neurodegeneration in the central nervous system. Increased inflammation results in cytotoxic effects, exacerbating symptoms of neurodegenerative diseases. Debilitating neuroinflammation is the root cause of neurodegenerative diseases, such as Parkinson’s disease, Alzheimer’s disease, amyotrophic lateral sclerosis, and multiple sclerosis, as well as autoimmune encephalomyelitis. Long-term inflammation of the nervous system causes the release of pro-inflammatory cytokines, chemokines, and reactive oxygen species by glial cells that mediate the mechanisms of neurodegenerative diseases (Kempuraj et al., 2017). One of the regulators of inflammation in the nervous system is the Peroxisome Proliferator-Activated Receptors (PPARs) signaling pathway. PPARs are transcription factors and are part of the nuclear hormone receptor superfamily. PARPs include PPARα (NR1C1), PPAR β/δ (NR1C2), and PPARγ (NR1C3). PPARγ plays an important role in the context of neuroinflammation, as it elicits the proliferation process, metabolism, differentiation, and development of the inflammatory response in the central nervous system. PPARγ is responsible for the inhibition of transcription factors such as protein activating transcription factors-1, Stat 1 NF-κB. PPARγ inhibits the expression of pro-inflammatory factors such as: COX-2, metalloproteinase-9 (MMP-9), A-scavenging receptor, iNOS and is involved in the production of pro-inflammatory cytokines. Thus, inhibition of PPARγ activity may contribute to chronic inflammation (Villapol, 2018). Kurt et al. (2020) observed that under LPS-induced inflammation in SIM-A9 microglia cells after GR24 treatment, there was a reduction in iNOS-dependent NO, with an IC_50_ value like that of the iNOS 1,400 W inhibitor. Moreover, a dose-dependent decrease in the expression level of iNOS and protein mRNA after incubation with GR24 was demonstrated. Furthermore, the use of GR24 against SIM-A9 microglia cells treated with LPS resulted in a significant decrease in the release of pro-inflammatory cytokines, i.e., TNF-α and IL-1β. The same effect was observed with TNF-α and IL-1β mRNA in bEnd.3 cells treated simultaneously with GR24 and LPS. The study also showed that the use of GR24 at a concentration of 5, 10, and 20 μM resulted in a strong reduction in COX-2 expression by 58%, 72%, and 78%, respectively. The researchers observed that LPS-induced inflammation in SIM-A9 cells suppressed PPARγ expression by about one-fifth, while GR24 attenuated this effect dose-dependently. The use of GR24 at a concentration of 20 µM restored the level of PPARγ expression to the control level. GR24 was also shown to induce the accumulation of NF-κB and Nrf2, which directly contributed to the increased expression of phase II detoxifying enzymes phase II like (NQO1) and (HO-1). These results allowed the consideration of a strigolactone analog as a potential therapeutic agent in neuroinflammation and neurodegenerative diseases.

Studies on the anti-inflammatory properties of strigolactone analogs were also conducted by Zheng et al. (2018). They used two optical isomers of GR24 (Figure 5). The MTT assay showed that the relative viability of RAW264.7 cells was greater than 99% at a concentration of GR24 isomers of 10 µM, indicating of these compounds did not have cytotoxicity. The level of release of inflammatory mediators, i.e., NO, TNFα and IL-6 after induction of LPS-induced inflammation in RA W264.7 cells was also determined. Two GR24 isomers significantly decrease the level of released pro-inflammatory factors. Zheng’s team also used an *in vivo* model in their study, damaged transgenic zebrafish larvae and investigated how GR24 isomers affect the migration of neutrophils and primitive macrophages, which are among the main types of cells associated with inflammation in the body. Traumatic inflammation was generated in zebrafish by clipping the tail. Fluorescence labeling generated an image where it was observed that the application of a dose of 10 and 20 µM of GR24 isomers significantly reduced the level of neutrophils and macrophages within the wound with time relative to the control group (truncation of the tail without treatment with compounds) (Zheng et al., 2018). Like the NF-κB pathway, the MAPK pathway is responsible for the signaling cascade that aims to modulate inflammation. There are several different MAPK signaling pathways, i.e., Extracellular Signal-regulated Kinases 1 and 2 (ERK1/2), c-Jun N-terminal kinases (JNK), p38^MAPK^, ERK5, ERK3/4, ERK7/8, and the Nemo -like Kinase (NLK). Modulation of the MAPK pathway is based on the cascade of mutual phosphorylation of kinases that activate subsequent kinases. Cytokine-activated kinases include ERK1 ERK2, while p38^MAPK^ and JNK are active after induction by environmental stress and mitogens (Moens et al., 2013). Zheng et al. (2018) observed that the exposure of RAW264.7 cells to LPS caused a significant increase in phosphorylated NF-κB, p65, IκBα, ERK1/2 and p38 MAPK. The addition of GR24 isomers to inflammatory RAW264.7 cells caused a concentration-dependent substantial depletion in the levels of phosphorylation of these factors. The research team showed that the anti-inflammatory effect of GR24 isomers is associated with inhibition of NF-κB and MAPK signaling pathways, which results in a reduction in the level of inflammatory mediators, that is, NO, TNF-α and IL-6 (Zheng et al., 2018). Table 2 shows that synthetic strigolactone analog GR24 is an efficient iNOS inhibitor and hinders NO release. Overproduction of NO is connected to many inflammatory diseases, immune-type diabetes, cancer, and more. Thus, the search for iNOS-specific inhibitors is highly needed.

**TABLE 2:** IC_50_ of GR24 on NO production after LPS-induced inflammation in animal(murine) cells.

Strigolactone analogs	Cell line	Stimulation time	IC_50_	References
GR24	RAW264.7 macrophage cells	24 h	5.56 µM	Tumer et al. (2018)
GR24	SIM-A9 microglia	24 h	6.2 µM	Kurt et al. (2020)
(R)-GR24	RAW264.7 macrophage cells	24 h	3.14 ± 0.24 µM	Zheng et al. (2018)
(S)-GR24	RAW264.7 macrophage cells	24 h	3.51 ± 0.22 µM	Zheng et al. (2018)

## 7 Antiviral properties of strigolactone analogs

Despite many scientific reports showing the promising properties of induce GR24 and other strigolactone analogs in anticancer therapy and in reducing inflammation, little is known about their antiviral properties.

Biolatti et al. (2020) tested the antiviral properties of strigolactone analogs against Human Cytomegalovirus (HCMV). In this investigation, the analogs of GR24, TH-EGO and EDOT-EGO (chemical structures presented in Figure 3) were investigated (Biolatti et al., 2020). The first step was to check whether the strigolactone analogs showed toxicity to normal cells. For this purpose, the human foreskin fibroblasts (HFF) cell line was used. The MTT test showed that the analogs used up to a concentration of 25 µM showed no cytotoxicity and cell viability was maintained at 90% after 144 h of incubation with the compounds. The next step was the evaluation of the antiviral properties of the tested analogs toward HCMV. TH-EGO and EDOT-EGO inhibited HCMV replication by more than 90% and were therefore selected for further analysis. An attachment and entry assay was performed to see how TH-EGO and EDOT-EGO affect the HCMV replication cycle. It was shown that both analogs did not have a negative effect on the virus attachment or penetration into cells, therefore, an analysis of the impact of these substances on HCMV gene expression was carried out. Western blot analysis found that its analogs interfered with the molecular events associated with the duplication of viral genetic material. This prompted researchers to evaluate the pro-apoptotic abilities of TH-EGO and EDOT-EGO. Annexin V-FITC staining showed that both analogs significantly contribute to the increase in the early apoptotic and necrotic cell population. The activity of caspase-3 was also assessed, which showed that both TH-EGO and EDOT-EGO promote the activity of this enzyme, both in infected and uninfected cells. Because the antiviral activity of strigolactone analogs has not been studied so far, it was decided to carry out *in silico* docking simulations. The results obtained on the HCMV Immediate-Early (IE1) Protein homology model allow us to conclude that the studied strigolactones may target anti-apoptotic proteins. However, further analyses are needed to confirm the originally obtained results (Biolatti et al., 2020).

The results presented in the above studies allow us to hypothesize that the GR24 strigolactone analog may also have antimicrobial and antiviral activity against human pathogens. However, the wide therapeutic potential of this analog may have tangible effects in the future.

## 8 Conclusion

Natural strigolactones and their synthetic analogs are molecules whose versatile action is their undeniable advantage. The development of research on these phytohormones makes it possible to discover their new properties and surprising biological activities in relation to many mammalian cells. This review presents in detail the latest research on the mechanisms of action of GR24 and its derivatives in terms of biomedical applications. The antioxidant capacity of SLs was indicated and several SLs activity pathways worth attention, according to the authors, were proposed in the schemes (Figures 4, 6–9). The most important data according to the type of effect are presented in the Table 3. In addition, it was mentioned that the biological properties of SL quite significantly depend on modifications in the A and D rings of the GR24 strigolactone, while the effect of modifications in the B ring is negligible.

**TABLE 3:** The effect of GR24 and GR24 analogs in mammalian cellular models.

Type of effect	Impact/Molecular pathways	Cells/Organs	GR24/GR24 analogs concentration	References
Anticancer	⁃ Reduction in growth	MCF-7	0.5–10 ppm (GR24)	Pollock et al. (2012)
	⁃ Increase in the apoptotic fraction of cells (sub-G1)	T47D		
	⁃ Disintegration of the mammosphere and reduced its growth	MDA-MB-231		
		MDA-436		
	⁃ Cell cycle arrest in the G2/M phase by inhibiting the expression of cyclin B	MCF-7	2–60 µM (EG5, EG9c (EGO10), ST357, ST362 and MEB55)	Pollock et al. (2014)
	⁃ Reducing the level of cyclin B, decreased the expression of Cdc25C phosphatase	MDA-MB-231		
	⁃ Increased expression and phosphorylation of JNK and p38 kinases with decrease in ERK1/2 kinase activity	MDA-MB-436		
	⁃ Reducing the expression of the ALDH1 marker			
	⁃ Inducing apoptosis	A172	1–100 µM (EGO10, IND)	Antika et al. (2022)
	⁃ G1 cell cycle arrest	U87		
	⁃ Inducing expression of Bax/caspase-3 genes			
	⁃ Downregulating Bcl-2 expression			
	⁃ Inducing apoptosis	HCT116	0.5–10 µM (Analog 6—Figure 5)	Yang et al. (2022)
	⁃ Increasing in the activity of caspase-3, caspase-8, and PARP1	SW620		
	⁃ Blocking cells in the S phase of the cycle			
	⁃ Inhibition of autophagy/mitophagy			
	⁃ Inhibition of tumour growth	HCT116 xenograft mouse model	50 mg, 100 mg (Analog 6—Figure 5)	
Anti-angiogenic	⁃ Disrupts the normal development of the chorioallantoic membrane	Chicken embryo	1, 10, and 25 nmol/CAM	Carrillo et al. (2019)
	⁃ Inhibits the formation of distal intersegmental vessels	Zebrafish	0.1–10 µM	
	⁃ Cell accumulation in the G2/M phase	HUVEC	1–100 µM (GR24)	
	⁃ Reduction of FAK activity by disrupting VEGF signalling	MDA-MB-231		
	⁃ Increasing the expression of VE-cadherin and PECAM-1	HeLa		
		SK-N-SH		
		U87MG		
		HT-1080		
		HepG2		
		HT-29		
		U2OS		
Antioxidant	⁃ Increasing the level of H_2_O_2_ and O_2_	Strigolactone-deficient rice mutants—d10-1 and d17-1	-	Mostofa et al. (2021)
	⁃ Higher level of MDA			
	⁃ Much lower activity of antioxidant enzymes (SOD APX GPX GR GST) compared to wild-type plants			
	⁃ Reducing the expression of genes responsible for the synthesis of antioxidant enzymes			
	⁃ Reducing the activity of non-enzymatic antioxidant - GSH			
	⁃ Increasing the activity of antioxidant enzymes (CAT, SOD, POD, APX)	Rape seed (*Brassica rapa* L.) seedlings in a low-temperature stress environment	0.1 μmol/L (GR24)	Zhang et al. (2020)
	⁃ Reduction in ROS production			
	⁃ Increasing the content of chlorophyll and carotenoids			
	⁃ Increased expression of antioxidant enzyme genes (CAT, SOD, APX, POD), NADPH, oxidase genes (RbohA ∼ D, RbohF-G), MAP kinase genes (MPK3, MPK6) and cold-related genes (COR, ICE1)			
	⁃ Increasing total level of chlorophyll	Cucumber seedlings (*Cucumis sativus* L.) in limited access to light	10 μM (GR24)	Zhou et al. (2022)
	⁃ Reducing the effects of light stress, and thus increasing the values of net photosynthetic rate, stomatal conductance, and transpiration rate			
	⁃ Stimulation of the expression of the genes of the antioxidant enzymes APX, GS, DHAR and NADH			
	⁃ Reduction in the expression of the RBOH gene			
	⁃ Reduction of the level of H_2_O_2_ and MDA			
	⁃ Increasing activity of sucrose synthase and the sucrose phosphate synthase level and increasing expression of genes encoding these enzymes			
	⁃ Improving organoleptic properties of fruit firmness, citrus color index, titratable acidity, total soluble solid, and soluble sugar content	Fruit (*Citrus sinensis* L.) were only stored at ambient temperature for 91 days	200 μmol/L (GR24)	Ma et al. (2022)
	⁃ Decreasing level of H_2_O_2_ and MDA			
	⁃ Increasing the level of antioxidants ascorbate-glutathione and the total phenol content			
	⁃ Inducing the activity of enzymes—CAT, GR, APX			
	⁃ Activation of Nrf2 signalling and increasing expression of downstream ARE-responsive genes	RAW264.7	5, 10, 20, 50, and 100 μM (GR24)	Tumer et al. (2018)
	⁃ Induction at the mRNA expression of the HO-1 and NQO1 enzymes	Hepa1c1c7		
	⁃ Disruption of the connection between KEAP1 and Nrf2			
	⁃ Activation of detoxifying enzymes phase II			
	⁃ Enhancement of the expression of antioxidant genes Nqo1 and heme oxygenase 1	Rat L6 myoblasts	60 µM (GR24)	Modi et al. (2018)
Inflammation modulation	⁃ Enhance the expression and activate SIRT1, increasing basal and insulin-stimulated glucose uptake	Rat L6 myoblasts	20, 60, and 100 μM (GR24)	Modi et al. (2017)
	⁃ Increasing the level of GLUT4 and stimulating its translocation			
	⁃ Stimulation biogenesis of mitochondria			
	⁃ Inhibition of the proliferation of cells	Mouse 3T3-L1 preadipocyte fibroblasts	20 and 60 μM (GR24)	Modi et al. (2018)
	⁃ Inhibition of fat accumulation during adipocyte differentiation			
	⁃ Activation of SIRT1 and increase of the level			
	⁃ Inhibition of adipogenesis through the suppression of PPARγ and C/EBPα			
	⁃ Reduction activity of NF-KB and interleukin 6			
	⁃ Inhibition of COX-2 expression	RAW264.7	5–100 μM (GR24)	Tumer et al. (2018)
	⁃ Inhibition release and expression of IL1β	C2C12 myoblasts		
	⁃ Reduction accumulation of p65 in the cell nucleus	Hepa1c1c7		
	⁃ Promotion of AKT activation in insulin resistant skeletal muscle cells			
	⁃ Downregulation expression of PEPCK and G6Pase			
	⁃ Reduction of iNOS-dependent NO	SIM-A9 bEnd.3	5, 10, and 20 μM (GR24)	Kurt et al. (2020)
	⁃ Decreasing in the expression level of iNOS and protein mRNA			
	⁃ Decrease in the release of TNF-α, IL-1β, TNF-α,			
	⁃ Reduction of COX-2 expression			
	⁃ Restoration of the level of PPARγ expression to the control level			
	⁃ Induction accumulation of NF-κB and Nrf2, which increased expression of detoxifying enzymes phase II (NQO1) and (HO-1)			
	⁃ Decreasing levels of NO, TNFα and IL-6	RAW264.7	1.25, 2.5, 5, and 10 µM (GR24 enantiomers)	Zheng et al. (2018)
	⁃ Increasing in the level of phosphorylated NF-κB p65, IκBα, ERK1/2 and p38 MAPK			
	⁃ Reduction in neutrophil and macrophage level of neutrophils and macrophages within the wound	Damaged zebrafish larvae	5, 10, and 20 µM (GR24 enantiomers)	
Anti-pathogenic	⁃			
	⁃ Interfering with the molecular events associated with the duplication of viral genetic material,	Human Cytomegalovirus (HCMV)	3–25 μM (TH-EGO, EDOT-EGO-Figure 5)	Biolatti et al. (2020)
	⁃ Increasing the early population of apoptotic and necrotic cells			
	⁃ Promote the activity of caspase 3			

The accumulation of evidence showed that plant hormones elicit a variety of responses in animal cells and may affect human/rodent physiology as stress responders. However, so many things including their presence in the human body, accumulation or synthesis in the brain, and modes of action remains mysteries especially for strigolactones, because of their quite new history as phytohormones. Therefore, it is worth paying attention to the fact that expanding knowledge within these phytohormones will not only contribute to the development of detailed knowledge on their cross-country effects but may also bring benefits resulting from the patenting of these compounds in medicine. Modification of glucose metabolism pathways is a very promising effect of strigolactones because diabetes is currently a lifestyle disease, especially type II diabetes associated with obesity. Blocking angiogenesis is one of the ways to inhibit tumor growth, but the drugs used have many troublesome and dangerous side effects, such as stroke or heart attack, and many others. The search for less toxic angiogenesis blockers remains a challenge. And finally, indication of the possibility of an antioxidant effect of SLs is of great importance in conjunction with the other properties of SLs reviewed in our paper.
