# Supplementary figures and images for: A multifaceted assessment of strigolactone GR24 and its derivatives: from anticancer and antidiabetic activities to antioxidant capacity and beyond
Source: Front Mol Biosci. 2023 Oct 26;10:1242935. doi: 10.3389/fmolb.2023.1242935 (PMC10639149; doi:10.3389/fmolb.2023.1242935)

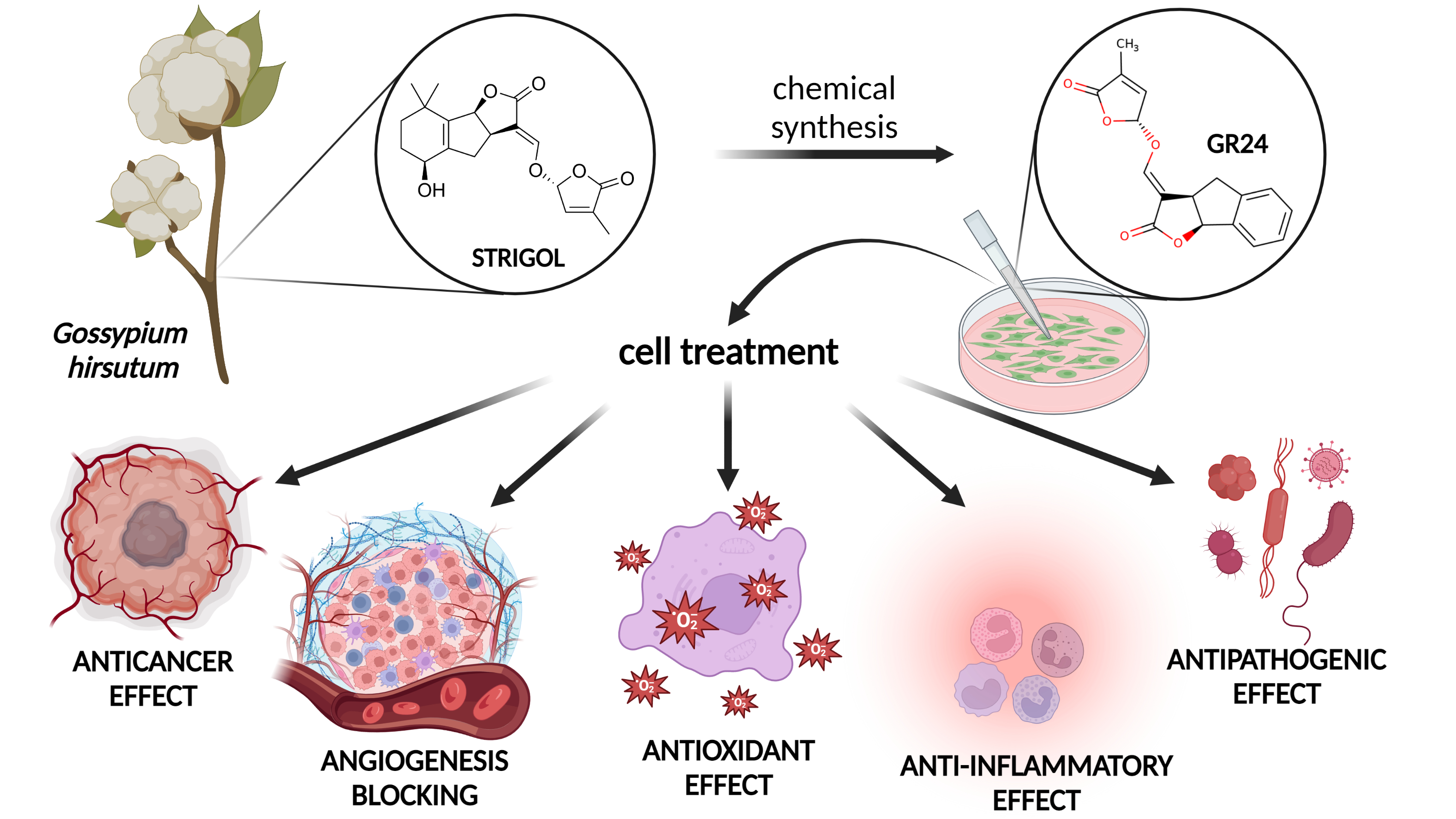

Supplement: Supplementary file 1 [file Image1.JPEG]
